# Parameter Evaluation and Application Demonstration of a New Type of Blood Vacuum Tube Cap Opening and Closing Machine

**Published:** 2019-09

**Authors:** Rong PU, Meicen PU, Qingsong XIN, Feng CHEN

**Affiliations:** 1. Department of Laboratory, Third People’s Hospital of Dongguan City, Dongguan 523000, P.R. China; 2. Department of Clinical Medicine, Fujian Medical University, Fuzhou 350000, P.R. China; 3. Department of Laboratory, Changan Hospital of Dongguan, Dongguan 523000, P.R. China; 4. Department of Laboratory, Guangji Hospital of Dongguan, Dongguan 523000, P.R. China

**Keywords:** Blood vacuum tube, Parameter estimation, Treatment

## Abstract

**Background:**

We aimed to evaluate the basic performance and basic parameters of a new type of blood vacuum tube cap opening and closing machine for further application.

**Methods:**

From July 2013 to March 2018, 110, 000 cases involved in the clinical trials in Third People’s Hospital of Dongguan City were selected as the instrument group, and 10,000 cases were selected as the manual operation group. The application demonstration and instrument performance assessment were performed by five units. Unified evaluation indicatory system and the standard of assessment were set up. The instrument assessments and demonstration tests were carried out by these 5 units. Finally, the basic parameters of the instrument were analyzed and compared.

**Results:**

The new instrument had excellent performance, and thirty parameters were excellent. Five patents had been granted already. There were no differences between the instrument groups in different units, and among different units. However, there were some differences in the manual operation group between different units. The average cap opening time was 21 “04 in the instrument group, and 152” 48 in the manual operation group (*P*<0.05); the cap closing time was 18 “56 in the instrument group, and 104”24 in the manual operation group (*P*<0.05). The instrument group outperformed the manual operation group in cap closing time, pollution rate, spill rate and failure rate (*P*<0.01). The design of the instrument was original.

**Conclusion:**

This blood vacuum tube cap opening and closing machine can perform a wide variety of functions, and it is stable, intelligent and superior to manual operation. More efforts need to be made for the industrialization of this instrument.

## Introduction

Cryopreservation of the blood is applied to determine cellular functions and is also a good source of DNA (1). However, massive collection and cryopreservation of the blood samples is labor- and time-consuming (2). Thus, integrated blood-collection automation is promising and extremely needed in laboratory (3–6).

The research and development of blood tube cap opening and closing machine is one of the key projects in laboratory. An intelligent approach is needed to design a blood tube cap opening and closing machine to save the manual labor and to safeguard against the biological risk (7–9). Although tube cap opening machines have been developed for the blood vacuum collection (10, 11), the efficiency of blood vacuum collection is still to be elevated. Therefore, automated lid opening and closing machine should be designed and invented, and such a machine will make blood vacuum collection a more time-saving and convenient progress (12, 13). Disk-type single-tube opening machines have appeared in USA, Korea and China, and the tube is uncapped one by one. Guangzhou Improve Medical Instruments Co., Ltd. develops the row-type uncapping machine, which cannot perform the capping operation.

Here we describe a new type of blood vacuum tube cap opening and closing machine, which can perform both the opening and closing operations and run stably. In addition, the tube caps can be retrieved one by one (14).

## Methods

### Grouping

From July 2013 to March 2018, 110, 000 cases involved in the clinical trials in Third People’s Hospital of Dongguan City were selected as the instrument group, and 10,000 cases were selected as the manual operation group. The application demonstration and instrument performance assessment were performed by five units. To protect confidentiality, units were denominated by English alphabets A to E.

The study was approved by the Ethics Committee of The Third People’s Hospital of Dongguan City. Signed written informed consents were obtained from the patients and/or guardians.

### Basic parameters

Thirty parameters were detected in application demonstration. Specifically, there were 15 basic performance indicators, including 5 structural parameters, 1 data parameter and 9 operational parameters; there were 15 clinical trial indicators, including 1 sample parameter, 2 time parameters, 6 failure parameters, and 6 biosafety parameters (Table 1).

**Table 1:** Basic performance indicators (30)

***Category***	***Number***	***Contents of parameters***
Data parameter	1	5 patents, 1 software copyright
Structural parameter	5	Touchscreen, alarm device, number of tubes accommodated, gripper, gripper cushion
Operational parameter	9	Self-checking, tube type selection, tube parameter adjustment, mode of motion of the clamping mechanism, dynamic gripping power, gripping range, gripping stability, instrument noise, instrument failure
Sample parameter	1	Number of samples in clinical trials
Time parameter	2	Average cap opening time, average cap closing time
Failure parameter	6	One-time cap opening failure rate, one-time cap closing failure rate, cap loss rate, rate of closing with the wrong cap, other failure rates, overall failure rate during the whole process of experiment
Biosafety parameter	6	Cap opening damage rate, cap closing damage rate, cap opening spill rate, cap closing spill rate, cap opening pollution rate, cap closing pollution rate

### Method of judgment

There were a total of 15 performance parameters, assessed on site by reading reference materials, on-site testing, and on-site operation.

### Assessment criteria of clinical trial indicators

There were 15 clinical trial indicators. The actual number of samples used in the experiment was referred. Time parameters were calculated as time-average number. Failure parameters consisted of cap opening and closing failures. Disordered placement of caps was considered as failure. If the cap was not tightly closed and could be removed with a single hand, then the cap closing operation was considered as failure. As to biosafety parameters, the presence of liquid outside the tube, in the instrument or gloves was judged as failure. The overall failure rate during the whole process of experiment was calculated as the sum of failure parameters, biosafety parameters, and cap retrieval parameters.

### Statistical process

SPSS 18.0 software (Chicago, IL, USA) was used for statistical analyses. Comparisons between the demonstration units and between the instrument group and manual operation group were performed using the *t*-test. *P*<0.05 indicated significant difference.

## Results

### Basic structure of the instrument

**Fig. 1:**
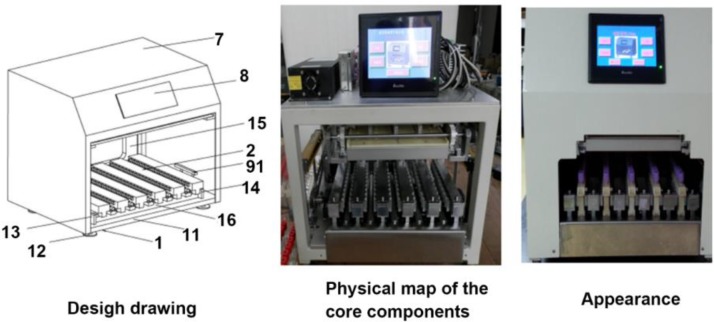
**A:** new type of blood vacuum cap opening and closing machine. Note 1. Mounting base; 11. Fixing and supporting transverse plate; 12. Leg of the mounting base; 13. Left supporting vertical plate; 14. Right supporting vertical plate; 15. Fixing and supporting vertical plate; 16. Transverse guide rail; 2. Gripping mechanism; 7. Sheet metal shell; 8. Touchscreen; 91. Photoelectric sensor

The instrument has an innovative design with simple appearance and complete functions. The instrument satisfies all five structural indicators. The instrument is equipped with a touchscreen and alarm device and can accommodate 50 tubes in one test. All 50 grippers were independent from each other, and each has cushion (Fig. 1).

### Core components

**Fig. 2:**
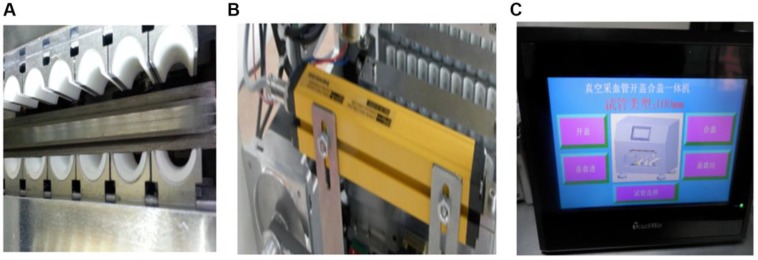
Physical map of core components. A: Gripping head. B: Alarm device. C: Touchsreen

Major innovations have been done to the gripping mechanism, sensing system, and touchscreen. The instrument can perform a wide variety of functions and run more stably and safely. A cushion pad is installed to the gripping head to make the gripping safer (Fig. 2A). An alarm device is equipped to give warning if there is any failure of cap opening, closing and movement, malpositioning or dysfunction of the mechanical components, and the presence of foreign bodies (Fig. 2B).

The touchscreen enables automatic tube parameter adjustment, execution of the cap opening and closing commands and operational monitoring (Fig. 2C).

### Operational parameters

The five units of application demonstration were generally informed of the basic performance of the instrument through patent searching, on-site testing and assessment. Five patents have been granted (ZL201610103496.4; ZL201610103497.9; ZL201310079727.9; ZL201320113773.1; ZL201620141509.2; ZL201620141510.5; ZL201620141511.X) alongside 1 software copyright (2016SR047305, control software for blood vacuum tube cap opening and closing machine V1.0). Self-checking that took 8 s indicated normal functioning of the instrument. The touchscreen and software provide an intelligent and easy operation. The gripping mechanism can make bi-directional movement, and the clamping force and dynamic force satisfy the requirements of clinical trials. The instrument has a low noise level, and the alarm device can give warning in case of failures.

### Results of clinical trials

The five demonstration units performed clinical trials with a total of 110,000 samples. These units obtained consistent one-time cap opening failure rate, one-time cap closing failure rate, cap opening damage rate, cap closing damage rate, cap loss rate, and the rate of closing with the wrong cap. However, other indicators varied across the five units, including average cap opening time, average cap closing time, cap closing spill rate, cap opening pollution rate, cap closing pollution rate, and overall failure rate of the whole process. Comparisons between the groups indicated no significant difference (Table 2).

**Table 2:** Results of clinical trials at five demonstration units

***Parameter***	***Unit***	***A***	***B***	***C***	***D***	***E***
***Total number of samples***	***Case***	***30000***	***20000***	***20000***	***20000***	***20000***
Average cap opening time of 50 tubes	s	20″57	21″18	20″52	21″25	20″46
Average cap closing time of 50 tubes	s	18″46	19″15	18″43	19″22	18″36
Cap opening spill rate	%	0.00	0.01	0.01	0.01	0.00
Cap closing spill rate	%	0.00	0.00	0.00	0.00	0.01
Cap opening pollution rate	%	0.01	0.01	0.01	0.01	0.00
Cap closing pollution rate	%	0.01	0.01	0.01	0.01	0.01
Overall failure rate of the whole process	%	0.02	0.03	0.03	0.03	0.02

### Results of manual operation

Manual cap opening and closing experiments were performed by the five units. The total number of samples was 10,000. The average tube damage rate was 0.00%. There were significant differences in cap opening time, cap closing time, failure rate, pollution rate, spill rate, cap loss rate and overall failure rate of the whole process, as shown in Table 3.

**Table 3:** Results of manual operation at the five units

***Parameter***	***Unit***	***A***	***B***	***C***	***D***	***E***
***Total number of samples***	***Case***	***6000***	***1000***	***1000***	***1000***	***1000***
Average cap opening time of 50 tubes	s	151″52	165″01	145″42	154″14	138″23
Average cap closing time of 50 tubes	s	102″18	110″44	98″57	105″27	90″58
One-time cap opening failure rate	%	2.85	1.23	3.12	2.98	3.12
One-time cap closing failure rate	%	3.20	1.12	3.68	2.68	3.85
Cap opening spill rate	%	1.86	0.56	2.56	1.56	2.88
Cap closing spill rate	%	0.01	0.01	0.03	0.01	0.04
Cap opening pollution rate	%	1.21	0.21	2.33	2.12	2.54
Cap closing pollution rate	%	1.83	0.33	2.22	0.74	2.65
Cap loss rate	%	1.51	0.56	1.91	0.91	2.83
Rate of closing with the wrong cap	%	1.61	0.25	2.23	0.81	3.82
Overall failure of the whole process	%	14.08	4.27	18.08	11.81	21.73

### Comparison between instrument group and manual operation group

A total of 120,000 samples were tested, including 110,000 in the instrument group and 10,000 in the manual operation group. The average cap opening time was 21 “04 in the instrument group, and 152” 48 in the manual operation group (*P*<0.05); the cap closing time was 18 “56 in the instrument group, and 104”24 in the manual operation group (*P*<0.05). The instrument group outperformed the manual operation group in cap closing time, pollution rate, spill rate and failure rate (*P*<0.01) (Table 4).

**Table 4:** Comparison of clinical trial results between the instrument group and manual operation group

***Parameter***	***Unit***	Average of the instrument group^*^	Average of the manual operation group^*^
Average cap opening time of 50 tubes	s	21″04	152″48
Average cap closing time of 50 tubes	s	18″56	104″24
One-time cap opening failure rate	%	0.00	2.66
One-time cap closing failure rate	%	0.00	2.91
Cap opening spill rate	%	0.01	1.89
Cap opening pollution rate	%	0.01	1.68
Cap closing pollution rate	%	0.01	1.55
Cap loss rate	%	0.00	1.54
Rate of closing with the wrong cap	%	0.00	1.74
Overall failure rate of the whole process	%	0.03	14.00

* *P*<0.05, comparison between the two groups

## Discussion

There are existing blood collection tube opening machines in the market, but there are no blood collection tube opening and closing machines (15–18). Our design is an original innovation in this aspect, by integrating laboratory medicine, bionics and informatics. The new type of blood collection tube opening and closing machine not only has optimized performance, but is also equipped with intelligent software. The human labor cost and the biosafety risk can be greatly reduced with this instrument.

Our team first developed the supporting system and optimized the core performance. Then we proceeded to develop the alarm system and intelligent software, and verify the instrument performance. Finally, application demonstration was carried out by 5 units using 120,000 tubes to assess the overall performance. This instrument is intelligent, highly efficient and safe. This instrument has four independent intellectual property rights; five patients (ZL201610103496.4; ZL201610103497.9; ZL201310079727.9; ZL201320113773.1; ZL201620141509.2; ZL201620141510.5; ZL201620141511.X) have been granted alongside one software copyright. Besides the advantages of integrated cap opening and closing operation and intelligent software, the touchscreen enables a free adjustment of tube parameters. The instrument is equipped with protective device and password system. The jacking parameters can be adjusted, and the instrument can give warning automatically and restore itself.

The instrument is stable and safe. Experiments carried out by the five units with a total of 110,000 samples indicated consistency in one-time cap opening failure rate, one-time cap closing failure rate, cap opening damage rate, cap closing damage rate, cap loss rate, and the rate of closing with the wrong cap. All these indicators were 0.00% across the five units. However, the results of other indicators varied, including average cap opening time, average cap closing time, cap closing spill rate, cap opening pollution rate, cap closing pollution rate, and overall failure rate of the whole process. Comparisons between the groups did not reveal significant differences. Thus the instrument has good stability and repeatability.

The instrument group was superior to the manual operation group in every indicator. Manual cap opening and closing operation fluctuate greatly, while the instrument is highly stable and safe. The five units performed manual operation experiments with 10,000 samples, and the tube damage rate was 0.00%. However, there were differences in cap opening time, cap closing time, failure rate, pollution rate, spill rate, cap loss rate and overall failure rate of the whole process. This is mainly due to the factors of cap opening and closing habits, speed and intensity of manual operating speed. For clinical trials, a total of 120,000 samples were tested at the five units, including 110,000 in the instrument group and 10,000 in the manual operation group. The average cap opening time was 21 “04 in the instrument group, and 152” 48 in the manual operation group; the average cap closing time was 18 “56 in the instrument group, and 104”24 in the manual operation group. The instrument group outperformed the manual operation group in cap closing time, pollution rate, spill rate and failure rate.

## Conclusion

This blood vacuum tube cap opening and closing machine can perform a wide variety of functions, and it is stable, intelligent and superior to manual operation. More efforts need to be made for the industrialization of this instrument.

## Ethical considerations

Ethical issues (Including plagiarism, informed consent, misconduct, data fabrication and/or falsification, double publication and/or submission, redundancy, etc.) have been completely observed by the authors.
